# Potential Clinical Value of Multiparametric PET in the Prediction of Alzheimer’s Disease Progression

**DOI:** 10.1371/journal.pone.0154406

**Published:** 2016-05-16

**Authors:** Xueqi Chen, Yun Zhou, Rongfu Wang, Haoyin Cao, Savina Reid, Rui Gao, Dong Han, Alzheimer’s Disease Neuroimaging Initiative

**Affiliations:** 1 Department of Nuclear Medicine, Peking University First Hospital, Beijing, China; 2 The Russell H. Morgan Department of Radiology and Radiological Science, Johns Hopkins University School of Medicine, Baltimore, Maryland, United States of America; 3 University Hospital, Hamburg-Eppendorf, Hamburg, Germany; 4 Department of Nuclear Medicine, the First Affiliated Hospital of Xian Jiaotong University, Xi'an, Shaanxi, China; 5 Department of Computer Science and Engineering, Oakland University, Rochester, Michigan, United States of America; Banner Alzheimer's Institute, UNITED STATES

## Abstract

**Objective:**

To evaluate the potential clinical value of quantitative functional FDG PET and pathological amyloid-β PET with cerebrospinal fluid (CSF) biomarkers and clinical assessments in the prediction of Alzheimer’s disease (AD) progression.

**Methods:**

We studied 82 subjects for up to 96 months (median = 84 months) in a longitudinal Alzheimer’s Disease Neuroimaging Initiative (ADNI) project. All preprocessed PET images were spatially normalized to standard Montreal Neurologic Institute space. Regions of interest (ROI) were defined on MRI template, and standard uptake values ratios (SUVRs) to the cerebellum for FDG and amyloid-β PET were calculated. Predictive values of single and multiparametric PET biomarkers with and without clinical assessments and CSF biomarkers for AD progression were evaluated using receiver operating characteristic (ROC) analysis and logistic regression model.

**Results:**

The posterior precuneus and cingulate SUVRs were identified for both FDG and amyloid-β PET in predicating progression in normal controls (NCs) and subjects with mild cognitive impairment **(**MCI). FDG parietal and lateral temporal SUVRs were suggested for monitoring NCs and MCI group progression, respectively. ^18^F-AV45 global cortex attained (78.6%, 74.5%, 75.4%) (sensitivity, specificity, accuracy) in predicting NC progression, which is comparable to the ^11^C-PiB global cortex SUVR’s in predicting MCI to AD. A logistic regression model to combine FDG parietal and posterior precuneus SUVR and Alzheimer’s Disease Assessment Scale-Cognitive (ADAS-Cog) Total Mod was identified in predicating NC progression with (80.0%, 94.9%, 93.9%) (sensitivity, specificity, accuracy). The selected model including FDG posterior cingulate SUVR, ADAS-Cog Total Mod, and Mini-Mental State Exam (MMSE) scores for predicating MCI to AD attained (96.4%, 81.2%, 83.6%) (sensitivity, specificity, accuracy). ^11^C-PiB medial temporal SUVR with MMSE significantly increased ^11^C-PiB PET AUC to 0.915 (p<0.05) in predicating MCI to AD with (77.8%, 90.4%, 88.5%) (sensitivity, specificity, accuracy).

**Conclusion:**

Quantitative FDG and ^11^C-PiB PET with clinical cognitive assessments significantly improved accuracy in the predication of AD progression.

## Introduction

Alzheimer’s disease (AD) is a slowly developed dementia. The symptoms could appear years after the biochemical changes in the brain happen. Paying considerable attention to the changes prior to clinical signs would be beneficial to both early diagnosis and possible treatment [1, 2]. People with mild cognitive impairment (MCI) proved to be at high risk of developing AD dementia, particularly for those in late MCI (LMCI) [3]. The pathological criteria for AD, or MCI due to AD, includes neuropathological evidence of neurofibrillary tangles and senile plaques with extracellular β-amyloid (Aβ) deposition and abnormal total tau (t-tau) or phosphorylated-tau (p-tau) deposition [4]. Although the clinical diagnosis of AD is mostly centered on the occurrence of clinical symptoms and cognitive impairment assessments, the new guideline proposed by National Institute of Aging and Alzheimer’s Association workgroups in 2011 provides updated details about the biomarkers associated with AD aside from clinical assessments [5].

Currently, the biomarkers of amyloidosis include Aβ and tau concentration in cerebrospinal fluid (CSF) and Aβ and tau brain deposition imaged by positron emission tomography (PET). Indicators extracted from structural and functional neuroimaging, such as atrophy detected by magnetic resonance imaging (MRI) and hypometabolism detected by ^18^F-fluorodeoxyglucose (FDG) PET, could also provide essential information closely associated with disease development [5]. The integration of these techniques brings new opportunities, as well as challenges, to the multimodality neuroimaging era in AD clinic and research [6].

FDG PET is used to detect the impairment of neuronal injury through the reduction of regional cerebral glucose metabolism in AD progression [7]. Amyloid deposition could also be measured by PET modality using tracers like ^18^F-florbetapir (^18^F-AV45) and ^11^C-Pittsbrugh Compound-B (^11^C-PiB). The correlation between the measurement of PET amyloid imaging and histological evidence of Aβ deposition were ascertained by several studies [8, 9].

It is now commonly accepted that the combination of different measurements yield promising evaluations for the prediction of disease progression. Longitudinal analysis of AD is essential because as AD develops over many years, the abnormality and order of changes for each biomarker are quite different [10, 11]. Nowadays, the quantitative PET technique is considered as a critical tool for monitoring and evaluating the AD progression. Evaluation of single or multiparametric PET performance in diagnosis and monitoring are indispensable for standardization and optimal use of PET in AD imaging. Through indirect study, comparable characteristics were found among the three widely-used radiotracers, FDG, ^18^F-AV45 and ^11^C-PiB [12]. However, the direct combination and comparison of these three radiotracers, especially for a longer follow-up time period, would still be meaningful for the further studies.

Alzheimer’s Disease Neuroimaging Initiative (ADNI) (http://adni.loni.ucla.edu/) is an international longitudinal multi-site multimodal AD imaging study with standardized image acquisition and processing procedures. In this study, a subpopulation with follow-up as long as 96 months from the ADNI project was selected to evaluate the potential clinical value of quantitative FDG, ^18^F-AV45 and ^11^C-PiB PET in the diagnosis and monitoring of AD progression. Various combinations of studying groups (normal controls, MCI, and AD), multiparametric PET images, CSF measurements, and clinical assessments were evaluated for improving the accuracy of diagnosis and monitoring of AD progression.

## Materials and Methods

### Data collection from ADNI

The anonymized and de-identified data used in the study were collected from ADNI database (adni.loni.usc.edu) by November 2014. The ADNI was launched in 2003 as a public-private partnership supported project. The ADNI data were collected from over 50 research sites and the ADNI study was approved by the local Institutional Review Boards (IRBs) of all participating sites, including our IRB at Johns Hopkins University and Albany Medical College, Banner Alzheimer’s Institute, Baylor College of Medicine etc. The detailed information and complete list of ADNI sites’ IRBs could be found at http://adni.loni.usc.edu/about/centers-cores/study-sites/ and http://www.adni-info.org/. Study subjects and if applicable, their legal representatives, gave written informed consent at the time of enrollment for imaging data, genetic sample collection and clinical questionnaires. The primary goal of ADNI is to test whether serial MRI, PET, other biological markers, and clinical and neuropsychological assessments can be combined to measure the progression of MCI and early AD. For up-to-date information, see www.adni-info.org.

A total of 82 ADNI subjects (Subject IDs listed in S1 File) were included in this study. Thirty-four and 48 subjects were diagnosed normal control (NC) and MCI at baseline, respectively. These subjects were followed for up to 96 months to ascertain the diagnosed status and progression (mean = 76.7 months; median = 84 months). To the best of our knowledge, this is a longest longitudinal study focusing on PET biomarkers from ADNI database.

All the subjects had baseline and follow-up FDG data. All ^18^F-AV45 and ^11^C-PiB PET scans for amyloid-β imaging were added in follow-up studies. Structural MRIs (1.5T or 3T, magnetization-prepared rapid acquisition gradient echo (MP-RAGE) were collected for each baseline and follow-up. Demographics, Apolipoprotein E (APOE) genotypes and CSF measurements, as well as clinical assessments were also downloaded from ADNI database.

### Status of subject: cognitively normal, MCI, and AD

The detailed criteria for each status and overall study protocol can be found at www.adni-info.org. In short, cognitively normal subjects had Mini-Mental State Exam (MMSE) scores between 24 and 30 inclusively, a Clinical Dementia Rating (CDR) of zero, were non-depressed, non-MCI, and non-demented. MCI subjects had MMSE scores between 24 and 30 (inclusive), a memory complaint, objective memory loss, a CDR score of 0.5, absence of significant impairment in other cognitive domains, and preserved activities of daily living. AD subjects presented with MMSE scores ranging from 20 to 26 inclusively, a CDR ≥ 0.5, and met the NINCDS/ADRDA criteria [13] for probable AD.

### Cognitive assessments

Besides MMSE, the cognitive assessments also included Alzheimer’s Disease Assessment Scale-Cognitive Sub-scale (ADAS-Cog). ADAS-Cog TOTAL 11 contains eleven items including word recall, recognition, naming, etc. (range 0–70) and ADAS-Cog Total Mod includes all the eleven items plus delayed word recall and number cancellation (range 0–85).

### CSF biomarkers: Aβ, t-tau, p-tau, t-tau/Aβ, p-tau/Aβ

CSF was acquired by lumbar puncture and the methods for collection of CSF samples are described at http://adni.loni.usc.edu/methods/documents/. The levels of CSF Aβ, t-tau, and p-tau were measured using the multiplex xMAP Luminex platform (Luminex Corp., Austin, TX) with Innogenetics (INNOBIA AlzBio3; Ghent, Belgium; for research-use only reagents) immunoassay kit-based reagent. The variables of CSF biomarker were Aβ, t-tau, p-tau, and ratios of t-tau/Aβ and p-tau/Aβ.

### Image acquisition, processing, and quantification

All FDG, ^18^F-AV45 and ^11^C-PiB PET scans were downloaded from http://adni.loni.usc.edu/ as the pre-processed format (co-registered, averaged, standardized image and voxel size, uniform resolution). The detailed methods could be found at http://adni.loni.usc.edu/methods-/pet-analysis/pre-processing/. Briefly, the separate PET frames were aligned to one another, averaged, reoriented and then interpolated into a standard image and voxel size (image volume 160×160×96, 1.5x1.5x1.5 mm in x, y, z). Lastly, all the PET images were smoothed to a uniform resolution of 8 mm in full width at half maximum (FWHM).

The downloaded PET and MRI images were then processed using Statistical Parametric Mapping software (SPM8, Wellcome Department of Imaging Neuroscience, London, United Kingdom) and MATLAB (The MathWorks Inc.). All preprocessed mean PET images were coregistered to structural MRI images at each follow up. The MRI images were normalized to standard Montreal Neurologic Institute (MNI) space using SPM8 with a MRI template provided by VBM8 toolbox [14, 15], and the transformation parameters determined by MRI spatial normalization were then applied to the coregistered PET images for PET spatial normalization. A total of 34 regions of interest (ROIs) were manually drawn on the MRI template using PMOD software (PMOD Technologies Ltd., Zürich, Switzerland) in standard MNI space. A global cortex was defined as a union of orbital frontal, prefrontal, superior frontal, lateral temporal, parietal, posterior precuneus, occipital, anterior cingulate, and posterior cingulate. The ROI of cerebellum gray matter was used as reference tissue, and the 34 ROIs including cerebellum were used as template ROIs for all subjects in the standard MNI space. Standard uptake value ratio (SUVR) images relative to the cerebellum ROI for ^18^F-FDG, ^18^F-AV45, and ^11^C-PiB were calculated in the MNI space (image volume: 121x145x121, voxel size: 1.5x1.5x15 mm in x, y, z). ROI SUVRs were obtained by applying ROIs to SUVR images.

### Statistical analyses

Receiver operating characteristic (ROC) analysis is commonly used to evaluate and optimize the performance of clinical diagnosis tests [16–18]. ROC analysis is a reliable statistical tool in the comparison and integrating quantitative multi-modal multi-parametric imaging of AD [19–26]. In the study, ROC analysis was used to evaluate the predictive value of each biomarkers separately for the disease progression in the NCs and MCI group. The highest area under the curve (AUC) and Youden index (Youden index = Sensitivity + Specificity—1) were used to select the cut-off value of biomarker’s measurement. The primary outcome was the diagnostic status of subjects from ADNI. In the NC group, the dichotomous variable indicated negative for those cognitively normal and positive for those converted to MCI or AD status. In the MCI group, the dichotomous variable indicated positive for those in MCI who converted to AD. In general, a test is acceptable in clinical efficacy if its AUC of ROC is not less than 0.70 [27–30].

First, the diagnostic values of FDG, ^18^F-AV45, and ^11^C-PiB in predicating AD progression were evaluated separately for each ROIs. In contrast to PET biomarkers, the accuracy of CSF biomarkers and clinical assessments for monitoring AD progression were also studied by ROC analysis. To investigate if multi-biomarker measurements improve the accuracy in monitoring the AD progression, a logistic regression model with stepwise regression was used to determine the optimal model to predict the disease progression. First, we tested the combination of cognitive assessments with SUVR of FDG or amyloid-β PET imaging, with or without CSF biomarkers in the logistic model. In this model, all the biomarker variables were collected at the same visit. Then the ^18^F-FDG data was combined either with ^18^F-AV45 or ^11^C-PiB to establish a prediction model for each group to discriminate the conversion.

Statistical analyses were carried out using IBM SPSS 21.0 and MedCalc 15.2.2. Statistical significance was set at p<0.05 and all tests were two-sided.

## Results

The demographic information and simple statistics of clinical assessments for all subjects at baseline visit are summarized in Table 1. During the study period, ten out of 34 NC subjects were converted to MCI or AD, and 24 out of 48 MCI subjects were converted to AD. In the NC group, there was no difference between converters and non-converters in age, gender, education, APOE carriers, and three clinical assessments at baseline. In the MCI group, in addition to the significant higher educations (p<0.05) in education years, the converters in MCI group had significant higher ADAS-cog TOTAL 11 (p<0.05) and ADAS-cog TOTMOD (p<0.01) scores than non-converters at baseline. We also tested all the regions of SUVR for FDG at baseline and found that none of the ROI SUVRs showed significant difference between converters and non-converters in both NCs and MCI group.

**Table 1:** Demographic and statistics of clinical assessments at baseline.

Variable	NC group (n = 34)		MCI group (n = 48)	
	NC convert (n = 10)	NC non-convert (n = 24)	MCI convert (n = 24)	MCI non-convert (n = 24)
	Mean (SD)	Mean (SD)	Mean (SD)	Mean (SD)
Demographic				
Male/ Female	9/1	17/7	14/10	20/4
Age	77.4 (3.4)	74.6 (4.0)	71.8 (7.0)	75.7 (7.1)
Education years	16.2 (4.1)	17.0 (3.0)	16.5 (2.7)	14.4 (2.8)*
APOE carriers (%)	40.0	16.7	54.2	45.8
Clinical Measures				
ADAS-cog TOTAL11	6.1 (1.4)	5.9 (2.9)	11.2 (4.2)	8.1 (2.8) **
ADAS-cog TOTALMOD	9.9 (2.2)	8.5 (3.6)	17.4 (7.2)	14.2 (4.8)*
MMSE	29.0 (0.9)	28.8 (1.5)	27.3 (1.8)	28.2 (1.3)
CDR	0	0	0.5	0.5

Note: NC: cognitively normal control (NC), MCI: mild cognitive impairment, ADAS-cog: Alzheimer’s Disease Assessment Scale-Cognitive Sub-scale, CDR: clinical dementia rating.

* p<0.05

** p<0.01.

The results of ROC analysis for each PET ROI SUVRs are summarized in Table 2. The FDG SUVRs of parietal, posterior cingulate, posterior precuneus, and caudate obtained significant prediction value for NC to MCI conversion (AUC>0.70). Among the 4 ROIs, the caudate had lowest specificity (48.8%) and accuracy (52.2%). The highest (specificity, accuracy) were attained by the posterior precunneus (87.4%, 85.7%), followed by parietal (83.3%, 82.2%), and posterior cingulate (79.5%, 79.1%). Most cortex ROIs of ^18^F-AV45 were identified for predicating NC to MCI conversion, and AUC of the global cortex was 0.748 with both high sensitivity (78.6%) and specificity (74.5%), and the corresponding cut off value was 1.288 (Table 2). The highest sensitivity of ^18^F-AV45 was attained in the parietal, posterior cingulate, and posterior precuneus (92.9%). The ventral striatum (VST) obtained the highest AUC (0.822) with (85.7%, 74.5%) (sensitivity, specificity). ^11^C-PiB was not included in Table 2 for NC group, because all of the initial ^11^C-PiB scans in the study were conducted on those NC non-converters (or NC converters but before conversion) and MCIs. For MCI to AD, the ROI FDG SUVRs of the posterior precuneus, posterior cingulate, and lateral temporal provided high specificity (72.5% to 81.3%) and accuracy (70.6% to 78.3%). For ^11^C-PiB ROI SUVRs, the medial temporal, orbital frontal, prefrontal, anterior cingulate, lateral temporal, amygdala, hippocampus, and putamen had AUC > 0.700 (Table 2). The highest sensitivities were obtained in the medial temporal and hippocampus (88.9%), followed by the global cortex (77.8%) with SUVR cut-off at 2.207. However, lower specificity and accuracy were also found in the medial temporal (57.4%, 61.9%) and hippocampus (50.0%, 47.6%). In contrast, the AUC values for all ^18^F-AV45 ROI SUVR were less than 0.700 with poor performance in specificity for predicating MCI conversion. The sensitivity and specificity of ^18^F-AV45 for the global cortex was (79.4%, 46.9%) and with as low as 0.612 of AUC. Note that the posterior precunneus, and posterior cingulate FDG were identified to have significant predicating values (AUC>0.72) for both NC to MCI and MCI to AD conversion. It is also worth noting that the parietal, posterior precunneus, and posterior cingulate SUVRs of both FDG and ^18^F-AV45 attained significant predicating values (AUC>0.72) for predicating NC to MCI conversion, and the lateral temporal SUVRs of both ^18^F-FDG and ^11^C-PiB were identified (AUC>0.70) for predicating MCI to AD conversion.

**Table 2:** PET biomarker- and ROI-specific efficacy in predicating Alzheimer’s disease progression.

Measurement	Number of converted (+)/ Non-converted (-) PET scans	AUC	95%CI	SUVR Mean±SD	SUVRcutoff	Sensitivity (%)	Specificity (%)	Accuracy (%)
**NC convert**								
FDG	15(+)/215(-)							
Parietal		0.804	0.747–0.853	(+):0.935±0.111; (-):1.067±0.153	0.978	66.7	83.3	82.2
PosCingulate		0.751	0.690–0.805	(+):1.258±0.223; (-):1.393±0.245	1.265	93.3	79.5	79.1
Caudate		0.739	0.677–0.794	(+):0.821±0.065; (-):0.942±0.195	0.928	100	48.8	52.2
PosPrecuneus		0.722	0.659–0.778	(+):1.279±0.250; (-):1.384±0.201	1.251	60.0	87.4	85.7
GlobalCortex		0.694	0.630–0.753	(+):1.064±0.155; (-):1.146±0.157	0.996	53.3	91.6	89.1
LatTemporal		0.617	0.551–0.680	(+):0.944±0.126; (-):0.997±0.114	0.877	53.3	89.3	87.0
^18^F-AV45	14(+)/47(-)							
VST		0.822	0.703–0.908	(+):1.519±0.275; (-):1.223±0.226	1.278	85.7	74.5	77.0
AntCingulate		0.772	0.647–0.870	(+):1.335±0.202; (-):1.131±0.226	1.183	85.7	74.5	77.0
PreFrontal		0.758	0.632–0.859	(+):1.527±0.242; (-):1.306±0.244	1.311	78.6	72.3	73.8
LatTemporal		0.754	0.627–0.855	(+):1.349±0.203; (-):1.178±0.188	1.246	78.6	78.7	78.7
PosPrecuneus		0.752	0.625–0.854	(+):1.583±0.275; (-):1.348±0.324	1.360	92.9	68.1	73.8
GlobalCortex		0.748	0.620–0.850	(+):1.447±0.203; (-):1.278±0.224	1.288	78.6	74.5	75.4
PosCingulate		0.739	0.610–0.843	(+):1.519±0.239; (-):1.324±0.310	1.329	92.9	63.8	70.4
ObiFronCo		0.737	0.609–0.842	(+):1.521±0.253; (-):1.307±0.236	1.551	64.3	85.1	80.3
Parietal		0.723	0.594–0.830	(+):1.304±0.217; (-):1.175±0.252	1.168	92.9	68.1	73.8
SupFronCo		0.722	0.592–0.829	(+):1.459±0.209; (-):1.296±0.237	1.318	78.6	72.3	73.8
Amygdala		0.705	0.575–0.815	(+):1.273±0.154; (-):1.157±0.158	1.125	85.7	48.9	57.4
Putamen		0.705	0.575–0.815	(+):1.612±0.210; (-):1.464±0.185	1.645	57.1	89.4	82.0
**MCI convert**								
FDG	59(+)/305(-)							
PosPrecuneus		0.742	0.694–0.786	(+):1.143±0.176; (-):1.293±0.152	1.174	62.7	81.3	78.3
PosCingulate		0.720	0.671–0.766	(+):1.158±0.154; (-):1.286±0.150	1.161	54.2	81.3	76.1
LatTemporal		0.706	0.656–0.752	(+):0.874±0.099; (-):0.951±0.095	0.899	61.0	72.5	70.6
Parietal		0.677	0.627–0.725	(+):0.937±0.141; (-):1.016±0.113	0.955	64.4	70.8	69.8
GlobalCortex		0.658	0.607–0.707	(+):1.037±0.098; (-):1.094±0.099	1.018	45.8	82.6	76.6
^11^C-PIB	9(+)/54(-)							
MedTemporal		0.759	0.635–0.858	(+):1.518±0.179; (-):1.348±0.187	1.362	88.9	57.4	61.9
ObiFronCo		0.743	0.617–0.845	(+):2.236±0.576; (-):1.718±0.651	2.207	77.8	68.5	69.8
Amygdala		0.733	0.606–0.836	(+):1.699±0.266; (-):1.481±0.266	1.530	77.8	64.8	66.7
Hippocampus		0.726	0.599–0.831	(+):1.646±0.188; (-):1.495±0.198	1.494	88.9	50.0	47.6
Putamen		0.726	0.599–0.831	(+):2.347±0.551; (-):1.929±0.500	2.456	66.7	83.3	81.0
AntCingulate		0.724	0.597–0.829	(+):2.248±0.611; (-):1.756±0.682	2.131	77.8	61.1	63.5
PreFrontal		0.708	0.580–0.816	(+):2.312±0.656; (-):1.803±0.702	2.172	77.8	63.0	65.1
LatTemporal		0.702	0.573–0.810	(+):2.045±0.541; (-):1.613±0.586	2.171	77.8	72.2	73.0
PosPrecuneus		0.698	0.569–0.807	(+):2.638±0.763; (-):2.082±0.817	2.549	77.8	70.4	71.5
PosCingulate		0.698	0.569–0.807	(+):2.640±0.772; (-):2.108±0.780	2.493	77.8	70.4	71.5
GlobalCortex		0.687	0.558–0.798	(+):2.148±0.514; (-):1.737±0.616	2.207	77.8	72.2	73.0
^18^F-AV45	34(+)/49(-)							
Parietal		0.645	0.532–0.747	(+):1.556±0.355; (-):1.444±0.353	1.257	73.5	53.1	61.5
SupFronCo		0.618	0.504–0.722	(+):1.609±0.400; (-):1.440±0.332	1.230	85.3	40.8	59.0
AntCingulate		0.616	0.503–0.721	(+):1.422±0.374; (-):1.287±0.329	1.117	82.4	44.9	60.3
GlobalCortex		0.612	0.498–0.717	(+):1.555±0.351; (-):1.420±0.325	1.285	79.4	46.9	60.2
Occipital		0.604	0.491–0.710	(+):1.550±0.304; (-):1.455±0.280	1.439	61.8	59.2	60.3
PosCingulate		0.598	0.484–0.704	(+):1.711±0.426; (-):1.567±0.416	1.328	85.3	44.9	61.4
PosPrecuneus		0.577	0.464–0.685	(+):1.725±0.470; (-):1.580±0.456	1.243	82.4	42.9	59.1

Note: ROI: region of interest; NC: cognitively normal control (NC), MCI: mild cognitive impairment, ADAS-cog: Alzheimer’s Disease Assessment Scale-Cognitive Sub-scale, PosCingulate: posterior cingulate; PosPrecuneus: posterior precuneus; VST: ventral striatum; AntCingulate: anterior cingulate; PreFrontal: prefrontal cortex; LatTemporal: lateral temporal cortex; GlobalCortex: union of orbital frontal, prefrontal, superior frontal, lateral temporal, parietal, posterior precuneus, occipital, anterior cingulate, and posterior cingulate; ObiFronCo: orbital frontal cortex; SupFronCo: superior frontal cortex; MedTemporal: medial temporal cortex.

As listed in Table 3 for ROC analysis of CSF biomarkers and clinical assessments, CSF Aβ showed highest AUC (0.850) with (100.0%, 82.1%) (sensitivity, specificity) for NC to MCI conversion. For MCI to AD, t-tau was the only significant CSF biomarker (AUC>0.70) with 93.3% sensitivity and 43.6% specificity. All three clinical assessments had poor sensitivity (42.9% to 53.3%) for predicating NC to MCI conversion, but they all attained high AUC (0.868 to 0.916), as well as sensitivity (83.6% to 86.2%) and specificity (84.1% to 85.8%) for predicating MCI to AD conversion.

**Table 3:** Diagnostic potential of CSF biomarkers and clinical assessments in predicating Alzheimer’s disease progression.

Measurement	AUC	95%CI	cutoff	Sensitivity (%)	Specificity (%)	Accuracy (%)
**NC convert**						
CSF						
Aβ	0.850	0.735–0.929	171.3 (pg/ml)	100	82.1	83.6
p-tau/ Aβ	0.725	0.590–0.836	0.145	100	60.8	64.0
t-tau/ Aβ	0.685	0.551–0.800	0.360	100	59.3	62.7
p-tau	0.604	0.464–0.732	24.2 (pg/ml)	100	47.1	51.8
t-tau	0.574	0.437–0.703	62.7 (pg/ml)	100	47.2	51.8
Cognitive						
ADAS-cog TOTALMOD	0.782	0.723–0.834	16.00	53.3	97.7	94.8
ADAS-cog TOTAL11	0.753	0.692–0.807	10.67	46.7	96.7	93.5
MMSE	0.690	0.625–0.750	27	42.9	92.0	89.0
**MCI convert**						
CSF						
t-tau	0.730	0.617–0.25	74 (pg/ml)	93.3	43.6	53.2
t-tau/ Aβ	0.667	0.552–0.769	1.078	43.7	87.3	78.5
p-tau	0.654	0.523–0.771	33 (pg/ml)	82.4	51.1	59.7
p-tau/ Aβ	0.644	0.513–0.762	0.168	88.2	40.0	53.2
Aβ	0.626	0.510–0.731	127.9 (pg/ml)	52.9	79.4	73.8
Cognitive						
MMSE	0.916	0.883–0.943	25.00	83.6	85.4	85.2
ADAS-cog TOTALMOD	0.887	0.850–0.918	22.67	86.2	85.8	85.8
ADAS-cog TOTAL11	0.868	0.829–0.901	13.67	84.5	84.1	84.2

Note: NC: cognitively normal control, MCI: mild cognitive impairment, ADAS-cog: Alzheimer’s Disease Assessment Scale-Cognitive Sub-scale, AUC: area under curve of receiver operating characteristic.

In the first logistic regression analysis of combined PET biomarkers and clinical assessments, the ROI SUVR of each three PET measurements (FDG, ^18^F-AV45 and ^11^C-PiB) was entered separately with the three clinical assessments, MMSE, ADAS-cog TOTAL11, and ADAS-cog TOTALMOD. The following three models were identified:

A: Logit(P) = 1.624+0.284*(ADAScogTOTALMOD)+7.832*FDG(PosPrecuneus)-17.957*FDG(Parietal)

B: Logit(P) = 15.467+0.084*(ADAScogTOTALMOD)-0.553*(MMSE)-3.950*FDG(PosCingulate)

C: Logit(P) = 3.847–0.634*(MMSE)+7.192*^11^C-PiB(MedTemporal).

**Fig 1:**
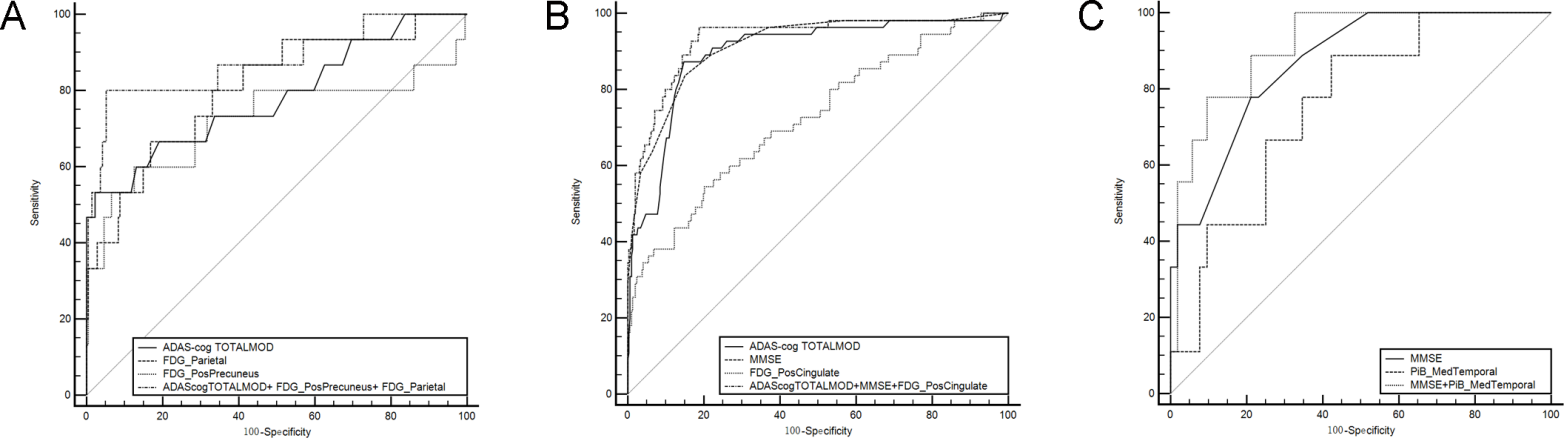
Receiver operating characteristic curves generated from logistic regression models of combined biomarkers for predicting conversions in cognitively normal control (NC) and mild cognitive impairment (MCI) groups. (A) ROCs of model A, ADAS-cog TOTALMOD, ^18^F-FDG SUVR of posterior precuneus and parietal for conversion from NC to MCI. (B) ROCs of model B, ADAS-cog TOTALMOD, MMSE and ^18^F-FDG SUVR of posterior cingulate for conversion from MCI to AD. (C) ROCs of model C, MMSE, and ^11^C-PiB SUVR of medial temporal for conversion from MCI to AD. The logistic regression models: Model A: Logit(P) = 1.624+0.284*(ADAScogTOTALMOD)+7.832*FDG(PosPrecuneus)-17.957*FDG(Parietal); Model B: Logit(P) = 15.467+0.084*(ADAScogTOTALMOD)-0.553*(MMSE)-3.950*FDG(PosCingulate); Model C: Logit(P) = 3.847–0.634*(MMSE)+7.192*^11^C-PiB (MedTemporal). PosCingulate: posterior cingulate; PosPrecuneus: posterior precuneus; MedTemporal: mesial temporal cortex.

Model A was selected for NC to MCI and AD conversion. Both model B and model C were selected for the prediction of MCI to AD conversion. The ROC curves of models A, B, and C are demonstrated in Fig 1, and the ROCs of each single PET biomarker and clinical assessments are also included in Fig 1A, 1B and 1C, respectively. The results of corresponding ROC analysis were summarized in Table 4. The (AUC, sensitivity, specificity) for model A, B, and C were (0.877, 80.0%, 94.9%), (0.932, 96.4%, 81.2%), and (0.915, 77.8%, 90.4%), respectively. The AUC of Model A was significantly higher than AUC of ADAS-cog TOTALMOD (p<0.01). Model B improved AUC significantly in contrast to each of its components for predication of MCI conversion (p<0.01 for ADAS-cog TOTALMOD and FDG posterior cingulate, and p<0.03 for MMSE). The AUC for Model C is only significantly higher than the AUC of ^11^C-PiB medial temporal SUVR. Adding the CSF concentration and APOE ε4 did not bring additional benefit to the stepwise logistic regression models.

**Table 4:** Diagnostic values of combined PET biomarkers and clinical assessments in predicating Alzheimer’s disease progression.

Variables	AUC	95%CI	Sensitivity (%)	Specificity (%)	Accuracy (%)	Compared with the combined variable	
						Z statistics	P level
**NC convert**							
ADAScogTOTALMOD	0.782	0.723–0.834	53.3	97.7	94.8	2.582	0.0098**
PosPrecuneus of FDG	0.722	0.659–0.779	60.0	87.4	85.6	1.324	0.1855
Parietal of FDG	0.804	0.747–0.854	66.7	83.3	82.2	0.860	0.3900
**Model A**	0.877	0.827–0.916	80.0	94.9	93.9		
**MCI convert**							
ADAScogTOTALMOD	0.898	0.861–0.927	86.2	85.4	85.5	2.587	0.0097**
MMSE	0.916	0.882–0.943	83.6	85.1	84.9	2.202	0.0277*
PosCingulate of FDG	0.726	0.676–0.772	54.4	79.9	75.7	5.127	<0.0001**
**Model B**	0.932	0.901–0.956	96.4	81.2	83.6		
MMSE	0.870	0.759–0.942	77.8	78.8	78.7	1.173	0.2409
MedTemporal of PiB	0.759	0.632–0.859	88.9	57.4	61.9	1.989	0.0467*
**Model C**	0.915	0.814–0.971	77.8	90.4	88.5		

Note: NC: cognitively normal control (NC), MCI: mild cognitive impairment, ADAS-cog: Alzheimer’s Disease Assessment Scale-Cognitive Sub-scale, AUC: area under curve, PosCingulate: posterior cingulate; PosPrecuneus: posterior precuneus. The 3 logistic regression models are listed below in detail: Model A: Logit(P) = 1.624+0.284*(ADAScogTOTALMOD)+7.832*FDG(PosPrecuneus)-17.957*FDG(Parietal); Model B: Logit(P) = 15.467+0.084*(ADAScogTOTALMOD)-0.553* (MMSE)-3.950*FDG (PosCingulate); Model C: Logit(P) = 3.847–0.634* (MMSE)+7.192*^11^C-PiB (MedTemporal).

* p<0.05

** p<0.01.

**Fig 2:**
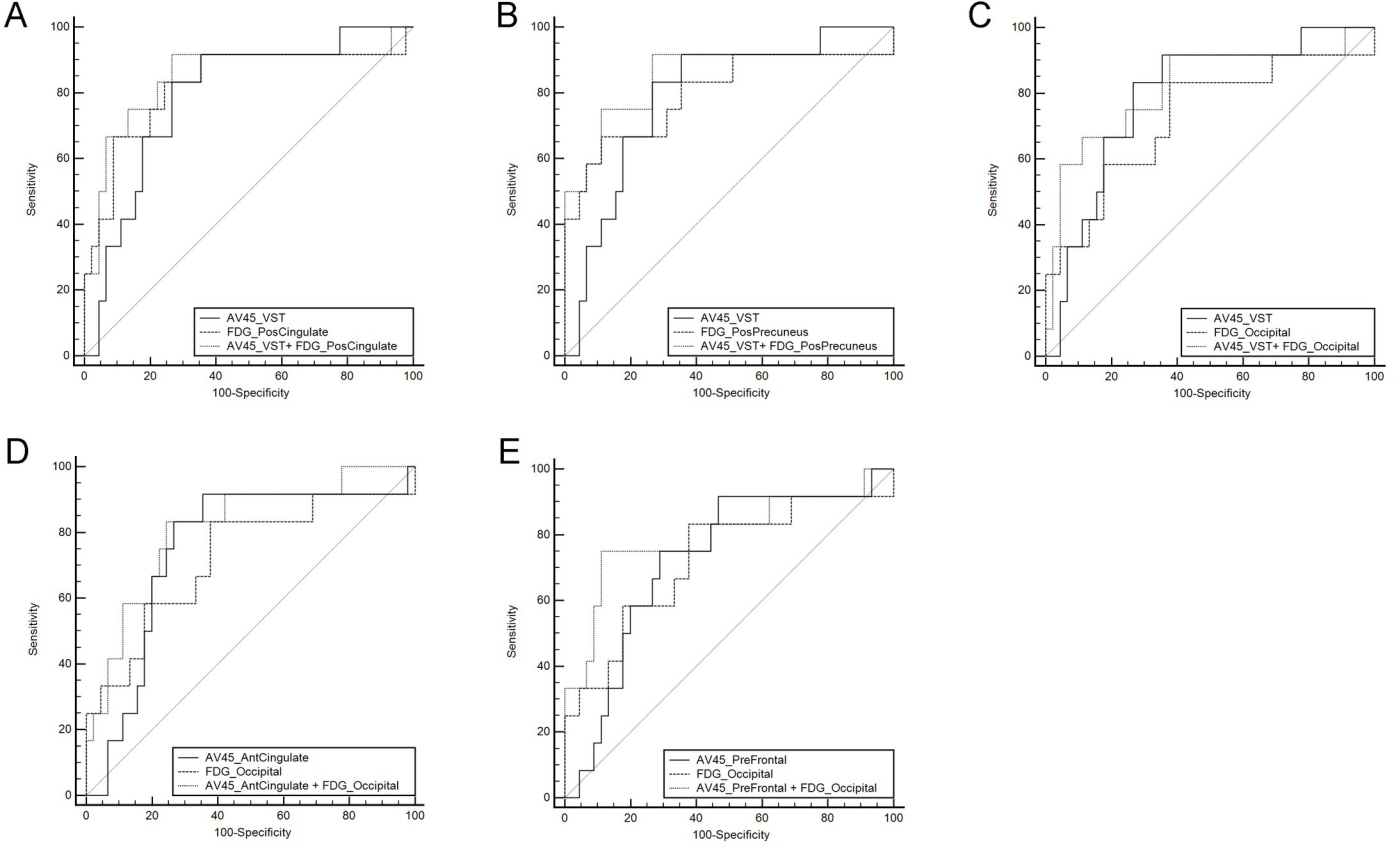
Receiver operating characteristic curves (ROCs) generated from logistic regression models of combined biomarkers of ^18^F-FDG and ^18^F-AV45 for predicting conversions in cognitively normal control subjects. (A) ROCs of model A, ^18^F-AV45(VST) and FDG(PosCingulate). (B) ROCs of model B, ^18^F-AV45(VST), and FDG(PosPrecuneus). (C) ROCs of model C, ^18^F-AV45(VST), and FDG(Occipital). (D) ROCs of model C, AV45(AntCingulate), and FDG(Occipital). (E) ROCs of model E, ^18^F-AV45(PreFrontal), and FDG(Occipital). The models A to E are expressed as below: Model A: Logit(P) = 6.580+2.724*^18^F-AV45(VST)-9.105* FDG(PosCingulate); Model B Logit(P) = 6.773+2.909*^18^F-AV45(VST)-9.332*FDG(PosPrecuneus); Model C: Logit(P) = 3.048+3.495*^18^F-AV45(VST)-7.959*FDG(Occipital); Model D: Logit(P) = 3.739+3.947*^18^F-AV45(AntCingulate)-8.735*FDG(Occipital); Model E: Logit(P) = 4.063+3.005*^18^F-AV45(PreFrontal)-8.468*FDG(Occipital). PosCingulate: posterior cingulate; PosPrecuneus: posterior precuneus; VST: ventral striatum; AntCingulate: anterior cingulate; PreFrontal: prefrontal cortex.

In the second logistic regression analysis, most of the scans we chose as pairs were conducted at the same visit (137 pairs of ^18^F-FDG and ^18^F-AV45: only four pairs had 1-year intervals 81 pairs of ^18^F-FDG and ^11^C-PiB: only two pairs had 1-year intervals). Five significantly improved (p<0.05) logistic regression models were identified for using ^18^F-FDG and ^18^F-AV45 to predicate NC conversion (Fig 2). However, the improvements of AUC in the 5 identified models were not statistically significant (p>0.05). When ^18^F-FDG combined with ^18^F-AV45 or ^11^C-PiB for predicating MCI conversion, neither was significant in the logistic regression model.

## Discussion

According to the current research, the decline of clinical function may appear years after the changes of PET imaging or CSF data [11, 31]. That means when the clues of conversion from imaging were observed, the clinical symptoms may not present at the same visit time. Taking this feature of progression into consideration, the longitudinal study was intended to accurately evaluate the value of biomarkers over time. Several studies also using ADNI data have been working on the predictive value of different measurements and achieving quite meaningful results [20, 32, 33]. Compared to a relatively short follow-up study [32], our study added more value in monitoring and predicting AD progression. Additionally, the inclusion of subjects in our study was based on the database with more available longitudinal PET imaging data, which was different from previous studies focusing on other biomarkers.

APOE ε4 is one of the most prominent genotypes in the onset of AD and has effects on other biomarkers like CSF levels of Aβ42 [34]. As an inherent genetic biomarker, the copies of APOE ε4 alleles do not differ from conversion vs. non-conversion at baseline in our study, which was similar with previous result [35]. However, the number of subjects with APOE ε4 carriers in the NC group was significantly lower than subjects in the MCI or AD group in the study (results not shown).

In the study, the cerebellum was chosen as the reference region for PET quantification, as it is commonly believed that FDG uptake in the cerebellum is not affected in MCI and AD, and that amyloid-β binding in the cerebellum is negligible in MCI and AD [36]. Quantitative FDG PET has been widely used in metabolic imaging of Alzheimer’s disease. Besides SUVR with conventional ROI determined by manually or templates, several other measurements, such as hypometabolic convergence index (HCI) [37, 38] and statistical-based clusters derived from FDG PET imaging, also helped in characterizing and predicting the AD progression. Regions associated with metabolic reduction in AD were mostly found in temporoparietal association cortices, and temporoparietal and posterior cingulate proved to be the target areas for diagnosis and monitoring AD progression [36, 39]. Hypometabolism of the posterior precuneus was also reported in several MCI conversion studies [40, 41]. Our results also clearly demonstrated that the parietal seemed to be the best indicator region in early phase of conversion, while posterior precuneus and cingulate were the regions with higher AUC and predictive value in MCI to AD, which were of potential clinical value for the diagnosis of AD progression.

For the amyloid imaging analysis, a high correlation between ^18^F-AV45 and ^11^C-PiB regions was confirmed by previous studies [12, 42]. Increased amyloid deposition was discovered in the frontal, temporal, parietal, cingulate, precuneus and striatum by other researchers [43, 44]. Note that the ability of these two tracers and their associated regions differed in our study: regions of ^11^C-PiB imaging acted effectively in distinguishing MCI converters from non-converters, and regions of ^18^F-AV45 imaging were sensitive to detect early stages of disease progression in the NC group. This could be due to the difference in studied population and scan time difference between the two tracers in the study.

In the study we demonstrated that ^11^C-PiB ROI SUVRs had high predictive value for MCI conversion to AD, which was consistent with other studies [19, 45]. It was reported that the best predicted region in ^11^C-PiB to discriminate AD from MCI was the lateral frontal cortex with an AUC of 0.86, 65% sensitivity and 75% specificity [19], which was higher than the ROI AUC values in our study. However, the limited 29 subjects and limited 2-year follow-up time should be taken into consideration when comparing the two studies [46].

In the ^18^F-AV45 imaging section of Table 2, more regions showed predictive significance in the NC progression. Among them, the VST indicated the highest AUC (0.822), sensitivity (85.7%) and specificity (74.5%). This was consistent with the results that amyloid deposition may begin in the striatum area [47, 48]. All ROI AUCs of ^18^F-AV45 for monitoring the MCI to AD progression were less than 0.7, which is usually considered as low predictive value in ROC analysis. Although there was still no conclusive opinion for the performance of predicting MCI conversion, several previous studies have highlighted the usefulness of ^18^F-AV45 in differentiating AD vs NC [49, 50].

From the single variable analysis of CSF biomarkers, CSF Aβ, and ratio of p-tau to Aβ worked well as predictors in the NC group, whilst CSF t-tau provided high sensitivity in predicating MCI conversion. However, in the logistic regression analysis, only PET biomarkers were selected in the models for prediction NC and MCI conversion. Note that our database was based on the PET imaging scans, and only about 60% of the subjects had CSF data. This may explain why the results did not improve significantly by adding CSF data, and CSF biomarkers were excluded in the final logistic model for progression. Previous studies showed that the multiparametric measurements with CSF information improved accuracy in predicating AD progression [33, 35].

The combined ADAS-cog, MMSE and FDG SUVR of the posterior cingulate was identified as the best multiparametric input model for MCI conversion with AUC of 0.932, and the improvement was more significant than any single input ROC analysis. In the second logistic regression analysis for studying the possible improvements, there was not any significant improvement in AUC when combining FDG with ^18^F-AV45 or ^11^C-PiB and single PET input. The improvements did not reach the statistical p value of 0.05, but it is worth further to be investigated in the ongoing project.

## Conclusions

In conclusion, ROC analysis with up to 96 months of longitudinal data identified ROI SUVRs of FDG PET for monitoring NC to MCI, and MCI to AD progression. ^18^F-AV45 is of significant prediction value for early diagnosis of AD, while ^11^C-PiB is suggested for monitoring the disease progression at late stage AD. Quantitative FDG and ^11^C-PiB PET with clinical cognitive assessments significantly improved accuracy in the predication of AD progression.

## Supporting Information

S1 File The 82 subject IDS. The list of 82 subject IDs used for downloading from ADNI database (http://adni.loni.usc.edu) were listed in S1 File.
(DOCX)

## References

[1] 2013 Alzheimer's disease facts and figures. Alzheimers Dement. 2013;9(2):208–45. 10.1016/j.jalz.2013.02.003 23507120

[2] Hebert LE, Weuve J, Scherr PA, Evans DA. Alzheimer disease in the United States (2010–2050) estimated using the 2010 census. Neurology. 2013;80(19):1778–83. 10.1212/WNL.0b013e31828726f5 23390181 PMC3719424

[3] Jessen F, Wolfsgruber S, Wiese B, Bickel H, Mosch E, Kaduszkiewicz H, et al AD dementia risk in late MCI, in early MCI, and in subjective memory impairment. Alzheimers Dement. 2014;10(1):76–83. 10.1016/j.jalz.2012.09.017 23375567

[4] Shaw LM, Vanderstichele H, Knapik-Czajka M, Clark CM, Aisen PS, Petersen RC, et al Cerebrospinal fluid biomarker signature in Alzheimer's disease neuroimaging initiative subjects. Ann Neurol. 2009;65(4):403–13. 10.1002/ana.21610 19296504 PMC2696350

[5] Albert MS, DeKosky ST, Dickson D, Dubois B, Feldman HH, Fox NC, et al The diagnosis of mild cognitive impairment due to Alzheimer's disease: recommendations from the National Institute on Aging-Alzheimer's Association workgroups on diagnostic guidelines for Alzheimer's disease. Alzheimers Dement. 2011;7(3):270–9. 10.1016/j.jalz.2011.03.008 21514249 PMC3312027

[6] Price JC. Molecular brain imaging in the multimodality era. J Cereb Blood Flow Metab. 2012;32(7):1377–92. 10.1038/jcbfm.2012.29 22434068 PMC3390805

[7] Herholz K, Carter SF, Jones M. Positron emission tomography imaging in dementia. Br J Radiol. 2007;80 Spec No 2:S160–7. 10.1259/bjr/97295129 18445746

[8] Ikonomovic MD, Klunk WE, Abrahamson EE, Mathis CA, Price JC, Tsopelas ND, et al Post-mortem correlates of in vivo PiB-PET amyloid imaging in a typical case of Alzheimer's disease. Brain. 2008;131(Pt 6):1630–45. 10.1093/brain/awn016 18339640 PMC2408940

[9] Choi SR, Schneider JA, Bennett DA, Beach TG, Bedell BJ, Zehntner SP, et al Correlation of amyloid PET ligand florbetapir F 18 binding with Abeta aggregation and neuritic plaque deposition in postmortem brain tissue. Alzheimer Dis Assoc Disord. 2012;26(1):8–16. 10.1097/WAD.0b013e31821300bc 22354138 PMC3286131

[10] Bateman RJ, Xiong C, Benzinger TL, Fagan AM, Goate A, Fox NC, et al Clinical and biomarker changes in dominantly inherited Alzheimer's disease. N Engl J Med. 2012;367(9):795–804. 10.1056/NEJMoa1202753 22784036 PMC3474597

[11] Jack CJ, Knopman DS, Jagust WJ, Petersen RC, Weiner MW, Aisen PS, et al Tracking pathophysiological processes in Alzheimer's disease: an updated hypothetical model of dynamic biomarkers. Lancet Neurol. 2013;12(2):207–16. 10.1016/S1474-4422(12)70291-0 23332364 PMC3622225

[12] Landau SM, Thomas BA, Thurfjell L, Schmidt M, Margolin R, Mintun M, et al Amyloid PET imaging in Alzheimer's disease: a comparison of three radiotracers. Eur J Nucl Med Mol Imaging. 2014;41(7):1398–407. 10.1007/s00259-014-2753-3 24647577 PMC4055504

[13] McKhann GM, Knopman DS, Chertkow H, Hyman BT, Jack CJ, Kawas CH, et al The diagnosis of dementia due to Alzheimer's disease: recommendations from the National Institute on Aging-Alzheimer's Association workgroups on diagnostic guidelines for Alzheimer's disease. Alzheimers Dement. 2011;7(3):263–9. 10.1016/j.jalz.2011.03.005 21514250 PMC3312024

[14] Ashburner J, Friston KJ. Unified segmentation. Neuroimage. 2005;26(3):839–51. 15955494 10.1016/j.neuroimage.2005.02.018

[15] Gaser C. Voxel based morphometry extension to SPM8. Available: http://www.neuro.uni-jena.de/vbm/2014/.

[16] Greiner M, Pfeiffer D, Smith RD. Principles and practical application of the receiver-operating characteristic analysis for diagnostic tests. Prev Vet Med. 2000;45(1–2):23–41. 10802332 10.1016/s0167-5877(00)00115-x

[17] Youden WJ. Index for rating diagnostic tests. Cancer. 1950;3(1):32–5. 15405679 10.1002/1097-0142(1950)3:1<32::aid-cncr2820030106>3.0.co;2-3

[18] Schisterman EF, Perkins NJ, Liu A, Bondell H. Optimal cut-point and its corresponding Youden Index to discriminate individuals using pooled blood samples. Epidemiology. 2005;16(1):73–81. 15613948 10.1097/01.ede.0000147512.81966.ba

[19] Bruck A, Virta JR, Koivunen J, Koikkalainen J, Scheinin NM, Helenius H, et al [11C]PIB, [18F]FDG and MR imaging in patients with mild cognitive impairment. Eur J Nucl Med Mol Imaging. 2013;40(10):1567–72. 10.1007/s00259-013-2478-8 23801168

[20] Gomar JJ, Conejero-Goldberg C, Davies P, Goldberg TE. Extension and refinement of the predictive value of different classes of markers in ADNI: four-year follow-up data. Alzheimers Dement. 2014;10(6):704–12. 10.1016/j.jalz.2013.11.009 24613706 PMC4416649

[21] Shaffer JL, Petrella JR, Sheldon FC, Choudhury KR, Calhoun VD, Coleman RE, et al Predicting cognitive decline in subjects at risk for Alzheimer disease by using combined cerebrospinal fluid, MR imaging, and PET biomarkers. Radiology. 2013;266(2):583–91. 10.1148/radiol.12120010 23232293 PMC3558874

[22] Ng S, Villemagne VL, Berlangieri S, Lee ST, Cherk M, Gong SJ, et al Visual assessment versus quantitative assessment of 11C-PIB PET and 18F-FDG PET for detection of Alzheimer's disease. J Nucl Med. 2007;48(4):547–52. 17401090 10.2967/jnumed.106.037762

[23] Leinonen V, Rinne JO, Wong DF, Wolk DA, Trojanowski JQ, Sherwin PF, et al Diagnostic effectiveness of quantitative [(1)(8)F]flutemetamol PET imaging for detection of fibrillar amyloid beta using cortical biopsy histopathology as the standard of truth in subjects with idiopathic normal pressure hydrocephalus. Acta Neuropathol Commun. 2014;2:46 10.1186/2051-5960-2-46 24755237 PMC4003513

[24] Herholz K, Evans R, Anton-Rodriguez J, Hinz R, Matthews JC. The effect of 18F-florbetapir dose reduction on region-based classification of cortical amyloid deposition. Eur J Nucl Med Mol Imaging. 2014;41(11):2144–9. 10.1007/s00259-014-2842-3 25002030

[25] Devanand DP, Mikhno A, Pelton GH, Cuasay K, Pradhaban G, Dileep KJ, et al Pittsburgh compound B (11C-PIB) and fluorodeoxyglucose (18 F-FDG) PET in patients with Alzheimer disease, mild cognitive impairment, and healthy controls. J Geriatr Psychiatry Neurol. 2010;23(3):185–98. 10.1177/0891988710363715 20430977 PMC3110668

[26] Walhovd KB, Fjell AM, Brewer J, McEvoy LK, Fennema-Notestine C, Hagler DJ, et al Combining MR imaging, positron-emission tomography, and CSF biomarkers in the diagnosis and prognosis of Alzheimer disease. AJNR Am J Neuroradiol. 2010;31(2):347–54. 10.3174/ajnr.A1809 20075088 PMC2821467

[27] Hosmer DW, Lemeshow S. Applied Logistic Regression 2nd Ed ed. New York: Wiley; 2000.

[28] Tan S, Kligerman S, Chen W, Lu M, Kim G, Feigenberg S, et al Spatial-temporal [(1)(8)F]FDG-PET features for predicting pathologic response of esophageal cancer to neoadjuvant chemoradiation therapy. Int J Radiat Oncol Biol Phys. 2013;85(5):1375–82. 10.1016/j.ijrobp.2012.10.017 23219566 PMC3606641

[29] Viccaro LJ, Perera S, Studenski SA. Is timed up and go better than gait speed in predicting health, function, and falls in older adults? J Am Geriatr Soc. 2011;59(5):887–92. 10.1111/j.1532-5415.2011.03336.x 21410448 PMC3522463

[30] Tokuhashi Y, Uei H, Oshima M, Ajiro Y. Scoring system for prediction of metastatic spine tumor prognosis. World J Orthop. 2014;5(3):262–71. 10.5312/wjo.v5.i3.262 25035829 PMC4095019

[31] Petersen RC, Roberts RO, Knopman DS, Boeve BF, Geda YE, Ivnik RJ, et al Mild cognitive impairment: ten years later. Arch Neurol. 2009;66(12):1447–55. 10.1001/archneurol.2009.266 20008648 PMC3081688

[32] Landau SM, Harvey D, Madison CM, Reiman EM, Foster NL, Aisen PS, et al Comparing predictors of conversion and decline in mild cognitive impairment. Neurology. 2010;75(3):230–8. 10.1212/WNL.0b013e3181e8e8b8 20592257 PMC2906178

[33] Dickerson BC, Wolk DA. Biomarker-based prediction of progression in MCI: Comparison of AD signature and hippocampal volume with spinal fluid amyloid-beta and tau. Front Aging Neurosci. 2013;5:55 10.3389/fnagi.2013.00055 24130528 PMC3795312

[34] Lautner R, Palmqvist S, Mattsson N, Andreasson U, Wallin A, Palsson E, et al Apolipoprotein E genotype and the diagnostic accuracy of cerebrospinal fluid biomarkers for Alzheimer disease. JAMA Psychiatry. 2014;71(10):1183–91. 10.1001/jamapsychiatry.2014.1060 25162367

[35] Da X, Toledo JB, Zee J, Wolk DA, Xie SX, Ou Y, et al Integration and relative value of biomarkers for prediction of MCI to AD progression: spatial patterns of brain atrophy, cognitive scores, APOE genotype and CSF biomarkers. Neuroimage Clin. 2014;4:164–73. 10.1016/j.nicl.2013.11.010 24371799 PMC3871290

[36] Herholz K. PET studies in dementia. Ann Nucl Med. 2003;17(2):79–89. 12790355 10.1007/BF02988444

[37] Chen K, Ayutyanont N, Langbaum JB, Fleisher AS, Reschke C, Lee W, et al Characterizing Alzheimer's disease using a hypometabolic convergence index. Neuroimage. 2011;56(1):52–60. 10.1016/j.neuroimage.2011.01.049 21276856 PMC3066300

[38] Schraml F, Chen K, Ayutyanont N, Auttawut R, Langbaum JB, Lee W, et al Association between an Alzheimer's Disease-Related Index and Gene Dose. PLoS One. 2013;8(6):e67163 23840615 10.1371/journal.pone.0067163 PMC3694066

[39] Herholz K. Cerebral glucose metabolism in preclinical and prodromal Alzheimer's disease. Expert Rev Neurother. 2010;10(11):1667–73. 10.1586/ern.10.136 20977325

[40] Clerici F, Del SA, Chiti A, Maggiore L, Lecchi M, Pomati S, et al Differences in hippocampal metabolism between amnestic and non-amnestic MCI subjects: automated FDG-PET image analysis. Q J Nucl Med Mol Imaging. 2009;53(6):646–57. 20016455

[41] Pagani M, Dessi B, Morbelli S, Brugnolo A, Salmaso D, Piccini A, et al MCI patients declining and not-declining at mid-term follow-up: FDG-PET findings. Curr Alzheimer Res. 2010;7(4):287–94. 19939228 10.2174/156720510791162368

[42] Wolk DA, Zhang Z, Boudhar S, Clark CM, Pontecorvo MJ, Arnold SE. Amyloid imaging in Alzheimer's disease: comparison of florbetapir and Pittsburgh compound-B positron emission tomography. J Neurol Neurosurg Psychiatry. 2012;83(9):923–6. 10.1136/jnnp-2012-302548 22791901 PMC4479493

[43] Wolk DA, Klunk W. Update on amyloid imaging: from healthy aging to Alzheimer's disease. Curr Neurol Neurosci Rep. 2009;9(5):345–52. 19664363 10.1007/s11910-009-0051-4 PMC2825106

[44] Jack CJ, Lowe VJ, Weigand SD, Wiste HJ, Senjem ML, Knopman DS, et al Serial PIB and MRI in normal, mild cognitive impairment and Alzheimer's disease: implications for sequence of pathological events in Alzheimer's disease. Brain. 2009;132(Pt 5):1355–65. 10.1093/brain/awp062 19339253 PMC2677798

[45] Hatashita S, Yamasaki H. Diagnosed mild cognitive impairment due to Alzheimer's disease with PET biomarkers of beta amyloid and neuronal dysfunction. PLoS One. 2013;8(6):e66877 10.1371/journal.pone.0066877 23799136 PMC3682994

[46] Chen WP, Samuraki M, Yanase D, Shima K, Takeda N, Ono K, et al Effect of sample size for normal database on diagnostic performance of brain FDG PET for the detection of Alzheimer's disease using automated image analysis. Nucl Med Commun. 2008;29(3):270–6. 10.1097/MNM.0b013e3282f3fa76 18349798

[47] Klunk WE, Price JC, Mathis CA, Tsopelas ND, Lopresti BJ, Ziolko SK, et al Amyloid deposition begins in the striatum of presenilin-1 mutation carriers from two unrelated pedigrees. J Neurosci. 2007;27(23):6174–84. 17553989 10.1523/JNEUROSCI.0730-07.2007 PMC3265970

[48] Selden N, Geula C, Hersh L, Mesulam MM. Human striatum: chemoarchitecture of the caudate nucleus, putamen and ventral striatum in health and Alzheimer's disease. Neuroscience. 1994;60(3):621–36. 7523983 10.1016/0306-4522(94)90491-x

[49] Joshi AD, Pontecorvo MJ, Clark CM, Carpenter AP, Jennings DL, Sadowsky CH, et al Performance characteristics of amyloid PET with florbetapir F 18 in patients with alzheimer's disease and cognitively normal subjects. J Nucl Med. 2012;53(3):378–84. 10.2967/jnumed.111.090340 22331215

[50] Barthel H, Gertz HJ, Dresel S, Peters O, Bartenstein P, Buerger K, et al Cerebral amyloid-beta PET with florbetaben (18F) in patients with Alzheimer's disease and healthy controls: a multicentre phase 2 diagnostic study. Lancet Neurol. 2011;10(5):424–35. 10.1016/S1474-4422(11)70077-1 21481640

