# Supplementary material for: Potential Clinical Value of Multiparametric PET in the Prediction of Alzheimer’s Disease Progression
Source: PLoS One. 2016 May 16;11(5):e0154406. doi: 10.1371/journal.pone.0154406 (PMC4868310; doi:10.1371/journal.pone.0154406)
Supplement: S1 File — The list of 82 subject IDs used for downloading from ADNI database (http://adni.loni.usc.edu) were listed in S1 File. (DOCX) [file pone.0154406.s001.docx]

**The 82 subject IDs.**

Data used in the study were collected from the Alzheimer’s Disease Neuroimaging Initiative (ADNI) database (adni.loni.usc.edu) by November 2014.

A total of 82 ADNI subjects were included in this study as listed below:

003_S_1074

003_S_1122

005_S_0546

005_S_0610

006_S_0498

006_S_1130

007_S_0101

009_S_0751

009_S_0842

009_S_1030

010_S_0419

011_S_0021

011_S_0023

013_S_1186

018_S_0055

018_S_0142

021_S_0626

022_S_0096

022_S_0130

024_S_0985

027_S_0074

027_S_0120

027_S_0408

029_S_1318

031_S_0294

031_S_0618

032_S_0214

033_S_0734

033_S_0741

033_S_0906

036_S_0945

037_S_0150

037_S_0377

037_S_0454

037_S_0552

037_S_0566

037_S_1078

041_S_0679

041_S_1418

052_S_1346

053_S_0919

057_S_0934

057_S_1007

072_S_0315

073_S_0746

099_S_0051

099_S_0291

099_S_0352

100_S_0047

114_S_0173

114_S_0378

114_S_0416

114_S_1106

114_S_1118

116_S_0361

126_S_0680

126_S_0709

127_S_0112

127_S_0259

127_S_0925

127_S_1427

128_S_0135

128_S_0200

128_S_0225

128_S_0227

128_S_0230

128_S_0272

128_S_0863

128_S_1043

129_S_0778

129_S_1246

130_S_0232

130_S_0285

130_S_0289

131_S_0123

137_S_0301

137_S_0722

137_S_0800

137_S_0972

137_S_0994

137_S_1414

941_S_1195
